# Relationship between urban green space and mental health in older adults: mediating role of relative deprivation, physical activity, and social trust

**DOI:** 10.3389/fpubh.2024.1442560

**Published:** 2024-08-29

**Authors:** Wen Zuo, Bin Cheng, Xinyan Feng, Xuefang Zhuang

**Affiliations:** ^1^School of Architecture and Urban Planning, Guangdong University of Technology, Guangzhou, China; ^2^School of Business, Macau University of Science and Technology, Taipa, Macao SAR, China

**Keywords:** urban green space, older adults, mental health, relative deprivation, China

## Abstract

**Introduction:**

The importance of improving older adults' mental health is increasing worldwide with the rapid development of the aging process. Green space is an important part of the urban built environment, demonstrates a deep connection with the mental health of older adults, and its internal mechanisms have been widely studied. This study analyzed the influence of urban green spaces on the mental health of older adults via three factors: relative deprivation, physical activity, and social trust.

**Methods:**

Based on the 2018 China Labor Dynamics Survey, a multi-level structural equation model was used to explore the mediating roles of relative deprivation, physical activity, social trust in urban green spaces, and the mental health of older adults.

**Results:**

Urban green space was positively correlated with the mental health of older adults. Relative deprivation and physical activities played a mediating role between urban green space and the mental health of older adults.

**Discussion:**

An increase in urban green spaces can help increase the number of older adults obtaining green space resources, and help them maintain good mental health. Secondly, older adults with a relatively homogeneous environment have more equal opportunities to obtain urban green space resources, which helps to reduce the comparison of older adults in access to green space resources and reduce the adverse impact of relative deprivation on their mental health. Additionally, increasing urban green spaces can encourage older adults to engage in physical activities and improve their mental health. Finally, we suggest improving the accessibility, fairness, and quality of green spaces, paying attention to the psychological needs of older adults, encouraging older adults to engage in physical activities in green spaces, and taking various measures to enhance the positive role of green space on the mental health of older adults.

## 1 Introduction

Population aging has become an irreversible trend in global population development. According to the World Social Report 2023, the number of people worldwide aged 65 years and over is expected to more than double by the middle of this century (1). China's population is gradually aging, and its older adult population is expected to account for ~25% of the total population by 2030; this increase is expected to be more evident in adults aged 80 years and older (2). In the context of urbanization with a large population gathering, the negative factors of the urban environment seriously affect the mental health of older adults. Research has shown that nearly two-fifths of older adults in China report subclinical levels of depression (3). Factors such as physical function, psychological distress, mental illness, and nursing relationships affect older adults' mental health (4–6). Considering the immense challenges brought about by aging, promoting healthy aging has become a countermeasure for China and the world to deal with the aging problem (7). The World Health Organization's definition of health refers not only to physical health, but also to a state of mental and social fulfillment. China's “14th Five-Year Plan for Healthy Aging” report highlighted that comprehensive and systematic intervention measures should be taken to better meet the health needs of older adults and build a healthy and livable urban and community environment, to improve the health level of older adults (7). As a scarce resource, urban green spaces play an important role in reducing the negative impacts of cities and improving the mental health of older adults (8). Therefore, understanding the influence mechanism of urban green spaces on the mental health of older adults is of great significance in building a healthy and livable city and promoting the development of healthy aging in China.

Mental health is the cornerstone of high-quality old-age care for older adults, and its influencing factors have been widely discussed by the academic community. Green spaces are an important factor affecting the mental health of older adults in urban environments through various biological, psychological, and social channels (9). Biologically, green space can reduce older adults' harmful environmental exposure by improving the urban environment, providing a livable urban environment, and improving older adults' mental health (10–13). Psychologically, attention recovery and stress reduction theories also suggest that green spaces can promote the mental health of older adults by reducing their psychological stress and improving negative emotions (14, 15). Socially, green spaces provide an open activity space for older adults, which helps them perform social interactions and physical activities to improve their mental health (16).

At the individual level of older adults, scholars have widely considered the role of the degree of deprivation of older adults on the relationship between green spaces and mental health. Among them, the relationship between absolute deprivation (that is, objective economic status) and green space has attracted the attention of most studies, such as the impact of socioeconomic status on accessibility (17) and fairness (18) of urban green spaces environment. Individuals with different degrees of absolute deprivation have different access to green space resources and, consequently, experience varied health benefits of green spaces (19). However, absolute deprivation pertains to measuring health inequality due to a gap in objective economic income; it is impossible to determine the relative income of different individuals in the group and the different effects on health brought about by this comparison (20). This perception of one being deprived of something compared with other individuals is called relative deprivation (21). Relative deprivation theory hypothesizes that various forms of socioeconomic comparisons lead to negative mental and physical health outcomes (22, 23). Moreover, most studies support the negative impact of relative deprivation on mental health. Relative deprivation has a greater negative impact on the health of older adults (24). Simultaneously, relative deprivation also affects physical activity, social trust, social cohesion, and other factors (14, 16, 25). Most studies support the link between relative deprivation and mental health as well as the link between urban greening and mental health. However, few studies have explored the relationship between relative deprivation and mental health in green spaces.

Research on the relationship between urban green spaces and older adults' mental health has gradually increased. However, more attention has been paid to the relationship between absolute deprivation and mental health, while ignoring the mediating role of individual physiological, psychological, and social factors, such as relative deprivation, physical activity, and social trust. Moreover, current studies have not sufficiently considered the impact path of these three factors on older adults' mental health. Therefore, using data from the 2018 China Labor Force Dynamics Survey and employing structural equation modeling (SEM), this study explored the mediating roles of relative deprivation, physical activity, and social trust in the relationship between green spaces and mental health in older adults, and proposed a theoretical framework to provide strategies for realizing healthy aging and building an aging-friendly and livable urban.

## 2 Literature review

### 2.1 Urban green space and mental health of older adults

The Third International Conference on Mental Health highlighted that mental health not only involves the absence of mental illness and good social adaptability, but also refers to the ideal personality and full development of spiritual potential, and the best mental state under certain objective conditions. Mental health levels are commonly assessed using several classic scales, including the General Health Questionnaire (26), Short Warwick Edinburgh Mental Health Scale (27), and EuroQol Five Dimensions Questionnaire (28). Roberts et al. (29) believe that people's mental health is influenced by three dimensions: physiological, psychological, and social factors. Physiologically, gender, education level, marital status, and other factors will affect the mental health of older adults (30–33). Psychologically, individuals who feel relatively deprived experience negative emotions, such as depression or stress, that may negatively impact mental health (34, 35). In the social dimension, social participation, social support, and relationships with family members are related to older adults' mental health status (36, 37).

As the most important settlement of people at present, the impact of urban construction and the social environment on people's mental health has been widely studied in academic circles, particularly the relationship between urban green spaces and mental health. Previous studies have shown that green spaces can reduce the negative impact of the urban environment on physical and mental health in various bio-psychosocial ways and can improve the mental health of residents (9). In terms of biological functioning, green spaces can affect air quality through particle deposition, dispersion, and modification, and reduce the negative impact of air pollution on residents' health and wellbeing (10). It also can adjust the urban climate, relieve the heat island effect, and reduce noise pollution, thereby promoting people's physical and mental health (11–13). By contrast, the psychological pathway mainly improves residents' mental health by reducing life pressure and arousing positive emotions. The attention recovery theory states that the interaction between residents and the surrounding green spaces can attract people's attention, mentally free up residents from daily troubles and problems, reduce pressure on residents, and improve their mental health (15). Meanwhile, the stress reduction theory states that the natural environment, as a restorative environment, can provide residents with opportunities to appreciate the natural landscape, generate positive emotions, and overcome negative thoughts, thus enhancing their ability to cope with stress and their mental health state (14). Studies have shown that older adults living in parks exhibit improved physical health, mood, and attention (38). The more green space visible, the lower the level of negative emotions people have (39). Moreover, green space is associated with the prevention of depression in older adults (40). From the social pathway, social participation and interaction are crucial for the relationship between green spaces and residents' mental health. First, residents living in natural environments can enhance their prosocial decisions and actions (such as cooperation, generosity, and trust), reduce antisocial behaviors (such as aggression and crime), and achieve good social health, which contributes to the maintenance of their psychological wellbeing (41). Second, neighborhood streetscape greening can indirectly affect the mental health of residents by promoting neighborhood attachment and community participation (14). There are also positive links between green Spaces, physical activity, and mental health. Liu et al. used SEM to study green space and walking behavior, stress, social cohesion, satisfaction with green space, and other factors. The results showed that most urban green spaces provided relatively safe and attractive outdoor physical activity spaces for people, which could attract people to engage in physical activities, stimulate the human body to produce natural feel-good hormones, and then improve mental health (42). In addition, green spaces help reduce the likelihood of depression. Studies have shown that physical activity, stress, and neighborhood social cohesion fully mediate the negative association between residential green space exposure and depression (43). Therefore, we propose the following hypothesis:

**Hypothesis 1: Green spaces directly impact older adults' mental health**.

### 2.2 Deprivation, green space, and mental health of older adults

Deprivation is a basic sociological concept that refers to a state of unsatisfied needs (44), and has two main divisions. On the one hand, objective economic deprivation, namely absolute deprivation, mainly refers to a situation in which some people's most basic life needs are not satisfied because of unfair treatment (45). On the other hand, it is the state of an individual's unsatisfied psychological needs, that is, relative deprivation. Specifically, it refers to the subjective perception of being deprived of one's interests by other groups when an individual is compared with other individuals with higher status and better living conditions around them, and the resulting feeling of a gap, anger, dissatisfaction, and other emotions brought about by such comparison (21). Social injustice is an important cause of relative deprivation (46).

Research on the role of deprivation in the relationship between green spaces and health has focused primarily on absolute deprivation. In the relationship between green space and absolute deprivation, the research results show that the urban green space resources available to individuals or groups are different due to different objective socio-economic status (47). In terms of green space accessibility, communities with higher degrees of absolute deprivation are further away from green spaces (48). Green space accessibility is more advantageous for residents in wealthier communities than in disadvantaged communities (49). Even if poor areas have equally accessible green spaces, the quality of their green spaces is relatively low (50). In terms of green space availability, low socio-economic status, and minority groups also have poor availability and quality of green spaces (51). Therefore, influenced by the relationship between absolute deprivation and green space, the health benefits of green spaces may be distributed unevenly among groups or communities with different socioeconomic statuses (19). Wang and Lan's (52) study showed that the accessibility and quality of park green space in socially disadvantaged communities are lower, health outcomes are poorer, and residents' access to park green space is associated with health outcomes. The health benefits of green spaces appear to be stronger for residents living in socioeconomic-advantaged neighborhoods than for residents in socioeconomic-disadvantaged neighborhoods (53). This inequality can be better explained by the theory of social causality: an individual's position in the social structure determines their level of health. Compared with people of higher absolute deprivation, those with lower absolute deprivation are more likely to have access to adequate material supplies, superior working conditions, and health services, and to have good physical and mental health (54, 55). Similarly, they are more likely to reap the health benefits of green spaces.

However, absolute deprivation can only study health inequality from the perspective of economic income gap; it is impossible to determine the different effects of comparison on health brought about by the relative income of various individuals in a group (20). The relative deprivation hypothesis explains the relationship between health inequality and income. It argues that inequality manifests through various forms of socioeconomic comparison (especially income inequality). These comparisons undermine social cohesion, social capital, trust, and wellbeing, ultimately leading to negative psychological and physical outcomes (22, 23). Theoretically, relative deprivation can affect health through two pathways. In the material pathway, relative deprivation limits individuals' access to things that represent a social standard of living, thereby adversely affecting their health. In the psychological pathway, inequality exacerbates negative emotions experienced by relatively poor people, leading to adverse health conditions (22). Numerous studies have shown that relative deprivation is detrimental to mental health. First, relative deprivation damages health-related quality of life, which is not conducive to the physical and mental health of residents (56). Simultaneously, relative deprivation significantly increases the risk of suicide in people over the age of 25 years (35). For the mental health of older adults, relative deprivation also has an important impact. Higher relative deprivation can damage the cognitive function and mental health of older adults. The negative effects are more pronounced on adults over 80 years of age and those living in urban areas than on middle-aged people (24). Liu et al. have also shown that increasing relative deprivation has a negative impact on the physical and mental health of older adults. The study also showed that older adults were more affected by relative deprivation than middle-aged people (57).

Additionally, relative deprivation is associated with physical activity and social trust. In terms of physical activity, relative deprivation was associated with lower physical activity. Studies have shown that the negative effects of relative deprivation may drive individuals to engage in unhealthy behaviors, including reduced physical activity and unbalanced eating habits (16). In studies of relative deprivation and obesity, it has also been shown that relative deprivation is associated with skipping breakfast, less physical activity, fewer healthy food choices, and a lower likelihood of dieting to lose weight (58). In terms of social trust, social psychology believes that people who feel relatively deprived may expand their psychological distance and distrust toward many members of society (59). Studies have shown that people of lower relative social status may feel distrust because they are unable to achieve the same status as people of higher relative status (60).

Generally, multiple empirical studies have shown that urban green spaces affect mental health through various pathways including physical activity and social trust (44, 61, 62). However, few studies have explored the role of relative deprivation in the relationship between green spaces and mental health. Relative deprivation, as an important factor affecting mental health, is associated with both physical activity and social trust. In addition, SEM provide a maximum-likelihood estimation of the entire system in a hypothesized model and enable the assessment of variables with the data (63). It allows for complex, multidimensional, and more precise analysis of empirical data taking into account different aspects of the examined reality and abstract concepts or theoretical constructs (64). As a multivariate data analysis tool, SEM is an important analytical means in mental health-related studies. For instance, Liu et al. (14) used the multilevel SEM model to study the relationship between natural outdoor environment and mental health, and Dzhambov et al. (65) also used SEM to study the relationship between residential perception of green space and residents' mental health. Therefore, in the internal influence mechanism of green space and the mental health of older adults, this study considered the indirect influence of relative deprivation, physical activity, and social trust. SEM was used to study the mediation effect, and the following hypotheses were put forward:

- **Hypothesis 2:** Relative deprivation, physical activity, and social trust play mediating roles in the relationship between green spaces and mental health of older adults.
- **Hypothesis 3:** Green spaces are related to physical activity and social trust through relative deprivation, which indirectly affects older adults' mental health.

## 3 Research design

### 3.1 Study area and population

Data were obtained from the 2018 China Labor Dynamics Survey (CLDS), a large-scale, nationally representative tracking survey of labor force dynamics designed and implemented by the Center for Social Science Research at Sun Yat-sen University. The 2018 CLDS contains data collected from 28 provinces in China, excluding Hong Kong, Macao, Taiwan, Tibet, Hainan, and Xinjiang. The database covers comprehensive data on 368 communities, 13,501 households, and 16,537 individuals in the labor force. The 2018 CLDS adopted a multi-stage, multi-level probability sampling method proportional to the size of the labor force, which minimizes sampling errors and ensures the randomness and scientific nature of sample selection. The present study drew on existing research (66), and defined older adults as individuals aged 60 years or older. We collected 2,465 valid samples from 119 Chinese cities.

### 3.2. Measurement of variables

#### 3.2.1. Mental health

The Center for Epidemiologic Studies Depression Scale was used to assess mental health. It contains 20 items used to assess depressive symptoms (67), scored on a four-point reverse scale (1 = almost always, or 5–7 days per week; 2 = often, or 3–4 days per week; 3 = rarely, or 1–2 days per week; and 4 = almost never, or < 1 day per week). The total score ranges from 20 to 80, with higher scores indicating improvement in older adults' mental health from the previous week. Cronbach's α of the mental health subscale was 0.946, indicating the reliability of the research questionnaire.

#### 3.2.2. Green spaces

Drawing on existing studies that have used the proportion of green space to measure urban green space (68), this study employed the green space coverage rates of built-up regions as indicators to quantify urban green space. The green space coverage rate of built-up areas refers to the proportion of urban built-up areas covered by greenery to the total built-up area, which was obtained from the 2018 China Urban Statistical Yearbook (69). Owing to the random processing of the community in the questionnaire, this study could not identify the community of each respondent, only the city information related to each respondent. Therefore, we could not study the accessibility of green spaces. This study assigned corresponding values to the respondents according to the green space coverage rate of each city.

#### 3.2.3. Relative deprivation

This study used the MacArthur scale to measure relative deprivation (70). Participants were asked to indicate their position in a self-defined society on a picture of an upright ladder with 10 rungs—with a score of “1” representing the bottom rung and a score of “10” representing the top rung. Relative deprivation was measured using the question, “Where do you currently see yourself in the hierarchy?” Higher scores indicate lower perceived relative deprivation.

#### 3.2.4. Social interaction

Drawing on the existing research literature, this study assessed the potential relationship between green space and mental health along social and behavioral dimensions (68). In the social dimension, social trust was evaluated through the following questions from the 2018 CLDS: “What is your level of trust in the following categories of people?” The categories included family members, relatives and friends, neighbors, classmates, fellow students, strangers, people who work or do things together, businesspeople who come into contact while buying things, and those who have religious beliefs. The trustworthiness score for social trust was determined using a five-point Likert scale (1 = not at all trustworthy; 5 = completely trustworthy). Total scores range from 9 to 45, with higher scores indicating a higher level of social trust in all types of people. Cronbach's α for social trust was 0.733, indicating that the questionnaire had good reliability. In the behavioral dimension, this study measured respondents' physical activity through the question, “Have you performed regular exercise in the last month?” and assigned values of 1 and 0, depending on whether the respondents answered yes or no, respectively.

#### 3.2.5. Covariates

This study adjusted for covariates of older adults' sociodemographic and individual health characteristics, and built environment (71). For individual-level covariates, this study included gender (binary variable: male vs. female), marital status (binary variable: not single vs. single), annual personal income (continuous variable), hukou (binary variable: local vs. non-local; i.e., whether the place of domicile was the same as their place of residence), and education (binary variable: educated vs. uneducated). For covariates of individual health characteristics, this study included illness and injury status (binary variable: no sickness or injury in the last 2 weeks vs. sickness and injury within the last 2 weeks), history of alcohol consumption (binary variable: no history of alcohol consumption vs. history of alcohol consumption), and history of smoking (binary variable: no history of smoking vs. history of smoking). Considering that the mental health of older adults may be affected by chronic diseases, as the 2018 CLDS does not have questions related to chronic diseases, the illness and injury status of older adults can be substituted for this question to some extent (8). For covariates of built environment, this study uses urban GDP per capita (continuous variable; i.e., gross urban product divided by total population) and urbanization rate (continuous variable; i.e., proportion of urban permanent population to the total permanent population), with data for the built environment taken from the 2018 China Statistical Yearbook (69).

### 3.3. Statistical analysis

**Figure 1:**
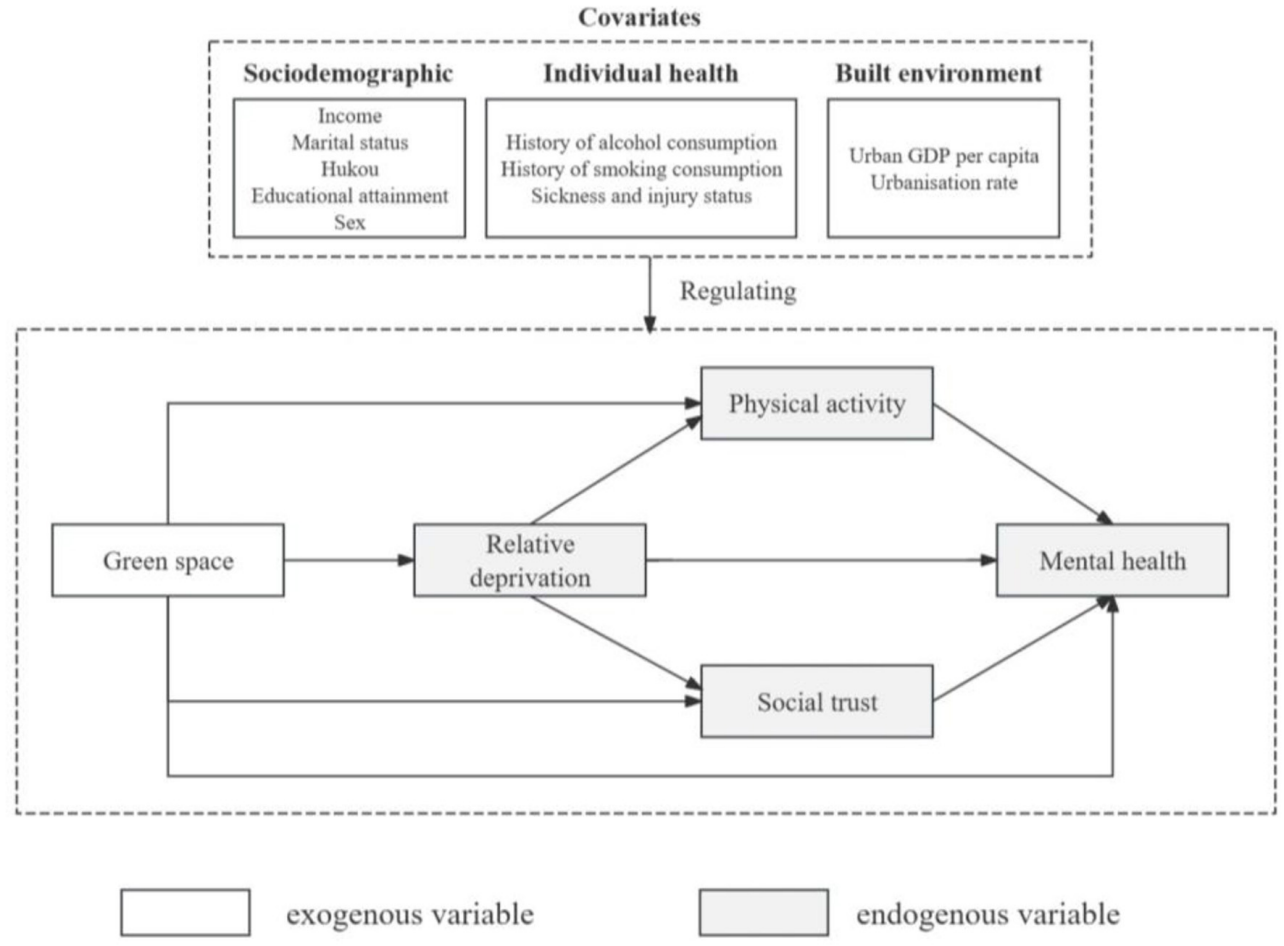
Theoretical framework for the SEM construction.

This study used SEM to examine the links between urban green spaces, latent media, and mental health. Notably, SEM can measure the total, direct, and indirect effects of one variable (e.g., urban green space) on another (e.g., mental health), allowing for the exploration of potential mechanisms behind the relationship between urban green space and mental health (72). In the SEM, chain mediation models were tested. Mental health was considered a continuous variable in the baseline model. Physical activity intensity was considered a binary variable. Relative deprivation and social trust were considered continuous variables. An analysis of this set of covariates was performed. The green space, sociodemographic, built environment, and individual health characteristics were set as exogenous variables. Relative deprivation, physical activity, social trust, and mental health were set as endogenous variables (Figure 1).

Because the variables estimated in this paper were observed variables rather than latent variables, the SEM without latent variables constructed in this paper can be expressed as follows:


(1) $$\begin{array}{l} y=By+\text{Γ}x+\delta \end{array}$$

where *y* refers to the N_Y_ × 1 vector of endogenous variables, *x* refers to the N_X_ × 1 vector of exogenous variables, *B* is the N_Y_ × N_X_ matrix of coefficients representing the direct effects of endogenous variables on other endogenous variables, Γ is the N_Y_ × N_X_ matrix of coefficients representing the direct effects of exogenous variables on endogenous variables, and δ is the N_Y_ × 1 vector of errors in the equation.

Additionally, this study considered existing research to determine the fit parameters for SEM (73), which tested the proposed models. The following model fit parameter criteria were used: the chi-square to degrees of freedom ratio (CMIN/DF) ≤ 5; root mean square error of approximation (RMSEA) ≤ 0.08; goodness-of-fit index (GFI) ≥0.90; normed fit index (NFI) ≥0.90; incremental fit index (IFI) ≥0.90; Tucker-Lewis index (TLI) ≥0.90; and comparative fit index (CFI) ≥0.90. SPSS Amos 26 was used for multi-level SEM, and STATA version 13.1 was used for basic pre-analysis data cleaning.

## 4 Results

### 4.1. Descriptive statistics

Table 1 presents the descriptive statistics for all the variables. The mean score of mental health was 71.73 (SD ±9.89), which was much higher than the cutoff (i.e., 2/3 of the total score of 80), revealing that the participants exhibited good mental health. The mean levels of relative deprivation and social trust were 4.50 (SD ±1.77) and 30.78 (SD ±4.12), respectively. Notably, the mean value of relative deprivation is less than half of the total relative deprivation score, indicating that older adults perceive themselves to be in a vulnerable position in society. Regarding physical activity, 72.5% of the older adults did not exercise regularly in the previous month, constituting the majority of the total sample and indicating a lack of physical activity among older adults. Regarding the control variables, 70.6% of the participants were educated, 90.2% were not single, 95.6% were local, 52.9% were male, 85% had no illness or injury in the preceding 2 weeks, 76.3% had no history of alcohol consumption, and 32.7% had no history of smoking. The mean annual income of older adults was 16,752.10 yuan (SD ± 18,911.34).

**Table 1:** Statistics of variables.

**Variables**	**Assignments**	**Total (*N* = 2,465)**
**Dependent variables**		
Mental health [mean (SD)]	Continuous variables (20–80)	71.73 (9.89)
**Independent variable**		
Green [mean (SD)]	Continuous variables	41.01 (3.60)
**Mediators**		
Relative deprivation [mean (SD)]	Continuous variables (1–10)	4.50 (1.77)
Social trust [mean (SD)]	Continuous variables (9–45)	30.78 (4.12)
Physical activity [*N* (%)]	1 = regular exercise for the last month	677 (27.5%)
	0 = no regular exercise for the last month	1,788 (72.5%)
**Covariates**		
Annual personal income [mean (SD)]	Continuous variables	16,752.10 (18,911.34)
Marital status [*N* (%)]	1 = non-single	2,223 (90.2%)
	0 = single	242 (9.8%)
Hukou [*N* (%)]	1 = local	2,357 (95.6%)
	0 = non-local	108 (4.4%)
Educational attainment [*N* (%)]	1 = educated	1,741 (70.6%)
	0 = uneducated	724 (29.4%)
Sex [*N* (%)]	1 = male	1,304 (52.9%)
	0 = female	1,161 (47.1%)
Sickness and injury status [*N* (%)]	1 = no sickness or injury within the last 2 weeks	2,096 (85.0%)
	0 = sickness and injury within the last 2 weeks	369 (15.0%)
History of alcohol consumption [*N* (%)]	1 = no history of alcohol consumption	1,881 (76.3%)
	0 = history of alcohol consumption	584 (23.7%)
History of smoking consumption [*N* (%)]	1 = no history of smoking consumption	806 (32.7%)
	0 = history of smoking consumption	1,659 (67.3%)

### 4.2. Structural equation analysis

#### 4.2.1. Analysis and fit of the model

**Figure 2:**
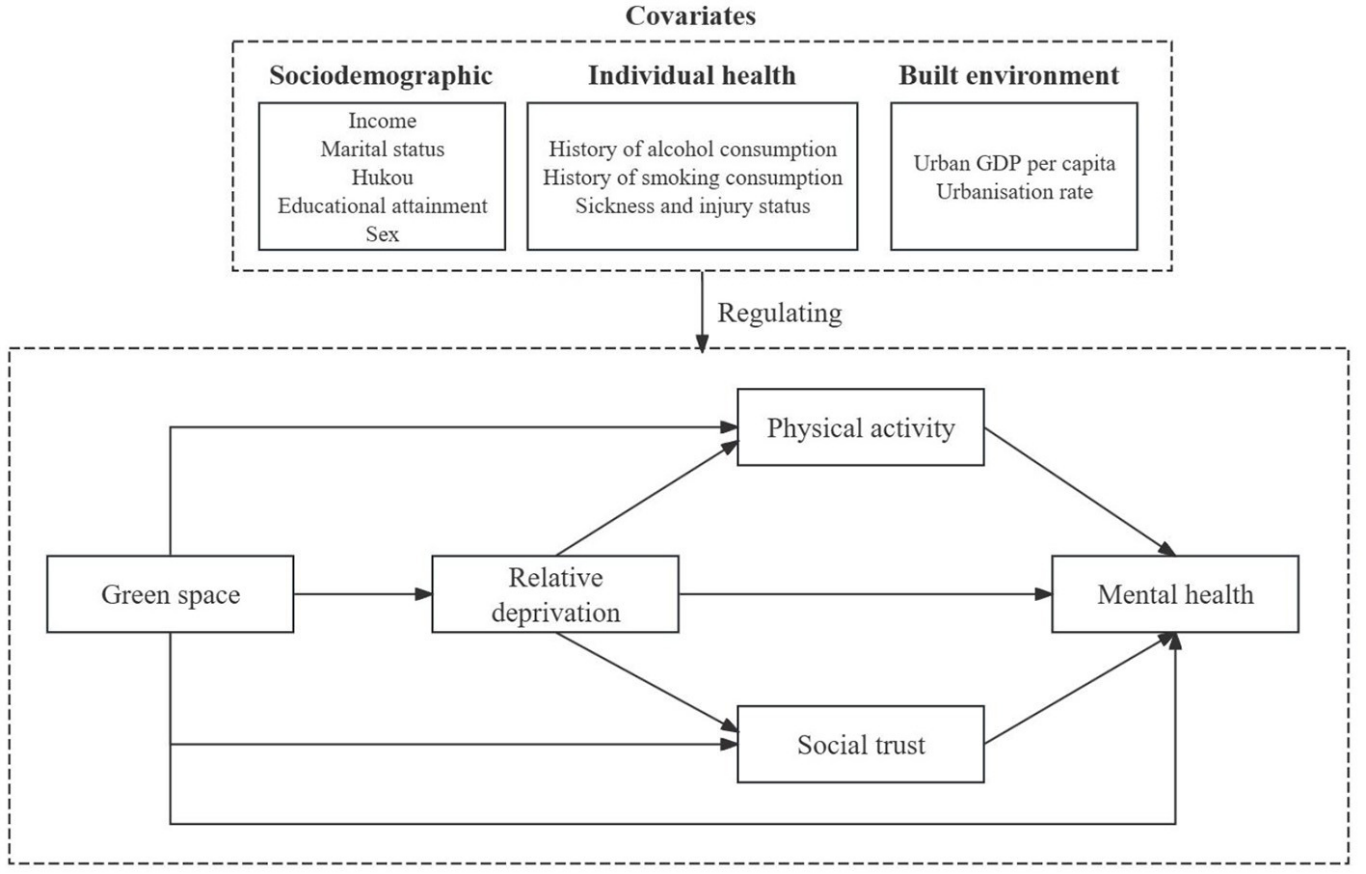
Conceptual framework.

SEM model was used to validate the conceptual framework (Figure 2). The fitness indicators of the model are as follows: CMIN/DF = 2.473, RMSEA = 0.025, GFI = 0.984, NFI = 0.969, IFI = 0.982, TLI = 0.969, and CFI = 0.981. The model passes the test for each indicator, as outlined in Section 3.3.

#### 4.2.2. Associations between green space, mediators, and mental health

**Figure 3:**
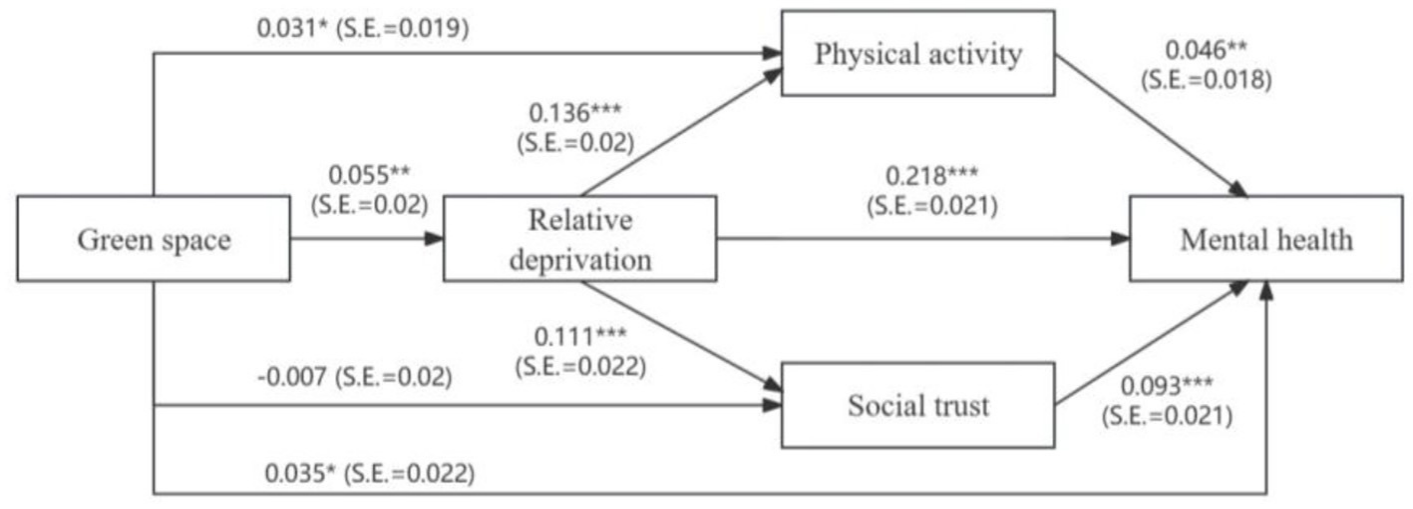
The model was adjusted for all covariates shown in Table 1. All the coefficients are standardized regression coefficients. *p < 0.1, **p < 0.05, ***p < 0.01.

The SEM model results are shown in Figure 3. Urban green space was significantly and positively associated with the mental health of older adults (β = 0.035, *p* < 0.1), indicating that an increase in urban green space favors the level of mental health of older adults. Therefore, Hypotheses 1 was supported. In the association between urban green space and mediating variables, urban green space was positively associated with both relative deprivation (β = 0.055, *p* < 0.05) and physical activity (β = 0.031, *p* < 0.1). The results reveal that an increase in urban green space allows more older adults to enjoy the benefits of green space resources in order to reduce the relative deprivation. In addition, the increase in green spaces provides more open space for older adults to engage in more physical activities. By contrast, there was no statistical relationship between urban green space and social trust (β = −0.007, *p* > 0.1). This suggests that an increase in urban green space does not directly increase social trust among older adults. Regarding the interrelationships of the three mediating variables, relative deprivation was positively associated with both increased physical activity (β = 0.136, *p* < 0.01) and social trust (β = 0.111, *p* < 0.01) among older adults. This suggests that lower relative deprivation among older adults increase their willingness to be physically active and social trusting. Regarding the relationship between the three mediating variables and mental health, older adults with lower relative deprivation were more likely to report higher levels of mental health (β = 0.218, *p* < 0.01). A decrease in relative deprivation suggests that older adults' perceived social status has risen, which to a certain extent enhances their psychological feelings of self-confidence, self-esteem, and pleasure, which is conducive to their psychological wellbeing. Older adults who engaged in regular physical activity were more likely to report higher levels of mental health (β = 0.046, *p* < 0.05). Additionally, older adults with higher levels of interpersonal trust in society were likely to have higher levels of mental health (β = 0.093, *p* < 0.01).

Table 2 presents the direct, indirect, and overall effects of urban green spaces and covariates on mental health. The overall effect of urban green spaces on mental health was statistically significant. The relationship between urban green spaces and mental health was mediated by relative deprivation and physical activity with indirect effect coefficients of 0.011 (*p* < 0.05) and 0.002 (*p* < 0.1), respectively. There is insufficient evidence in this study that social trust mediates the relationship between urban green spaces and mental health (β = −0.001, *p* > 0.1). Therefore, Hypotheses 2 was partially supported. This result reveals the role of urban green spaces in influencing mental health, mainly by reducing relative deprivation and promoting physical activity in older adults, thus improving mental health. In addition, urban green spaces can influence the level of mental health of older adults by influencing the feeling of relative deprivation, which in turn affects physical activity and social trust, with indirect effect coefficients of 0.001 (*p* < 0.05) and 0.001 (*p* < 0.05), respectively. Therefore, Hypotheses 3 was supported. These results reveal that a sense of relative deprivation plays an important mediating role between urban green spaces and mental health, which may, in turn, affect mental health through social interactions and other pathways.

**Table 2:** The direct effect, indirect effect and total effect of green space and covariates on mental health.

	**Direct effect Coef. (SE)**	**Indirect effect Coef. (SE)**	**Total effect Coef. (SE)**
Green space → mental health	0.035^*^ (SE = 0.022)	0.014^**^ (SE = 0.006)	0.049^**^ (SE = 0.022)
Green space → relative deprivation → mental health		0.011^**^ (SE = 0.005)	
Green space → physical activity → mental health		0.002^*^ (SE = 0.001)	
Green space → social trust → mental health		−0.001 (SE = 0.002)	
Green space → relative deprivation → physical activity → mental health		0.001^**^ (SE = 0.001)	
Green space → relative deprivation → social trust → mental health		0.001^**^ (SE = 0.001)	
**Covariates**			
Annual personal income → mental health	0.016 (SE = 0.019)		
Marital status → mental health	0.006 (SE = 0.019)		
Hukou → mental health	−0.007 (SE = 0.016)		
Educational attainment → mental health	0.046^**^ (SE = 0.022)		
Sex → mental health	0.100^***^ (SE = 0.025)		
Sickness and injury status → mental health	0.158^***^ (SE = 0.022)		
History of alcohol consumption → mental health	−0.001 (SE = 0.020)		
History of smoking consumption → mental health	0.037 (SE = 0.023)		
Urban GDP per capita → mental health	0.179^***^ (SE = 0.032)		
Urbanization rate → mental health	−0.067^**^ (SE = 0.035)		

Coef., coefficient; SE, standard error.

^*^p < 0.1.

^**^p < 0.05.

^***^p < 0.01.

The model was adjusted for all covariates shown in this Table. All the coefficients are standardized regression coefficients.

## 5 Discussion

### 5.1 Green spaces and mental health

The results of this study showed that older adults with more green spaces in their living environments exhibited better mental health than those with less green spaces. This finding is consistent with those of most existing evidence (74, 75). Natural landscapes within green spaces can provide a rich sensory experience that helps residents relieve stress, generate positive attitudes, and relieve negative emotions. This study takes urban green space coverage as a quantitative index, and the results also prove that urban green space coverage is related to the mental health of older adults. A possible explanation is the relationship between green space and urban environmental improvement. The improvement of urban greening coverage is beneficial to reducing air pollution in urban (76). Many studies have proved that air pollution in urban is one of the important causes of damage to the mental health of older adults (77–80), including the increased risk of depression (78), the occurrence of sleep disorders (80), and the decline of cognitive function (79). Moreover, older adults who exhibit reduced abilities due to physical or cognitive decline may be highly sensitive to changes in environmental factors (81). Such that urban green spaces may have better direct benefits for the mental health of older adults compared to residents of other age groups (82). It shows that the improvement of urban green space coverage helps maintain a good psychological state.

Land measurements may influence the association between green spaces and mental health in older adults. This study used the coverage rate of green space in the urban built environment as an indicator to quantify urban green space, while other relevant indicators of urban green space, such as type, quality, and subjective perception, were missing (83). For example, Wang et al. (84) evaluated the quality of green space with respect to accessibility, maintenance, change, naturalness, color, clear arrangement, shelter, presence of garbage, safety, and overall impression, and found that abundant green space is correlated with enhanced mental health. Regarding subjective perception, previous studies have shown that green spaces directly impact older adults' mental health through the perception of bodily organs, wherein streetscape greenery can directly affect older adults' mental health through their visual system (14).

### 5.2 Potential associations between green spaces and mental health

The relationship between green spaces and older adults' mental health is influenced by many complex and potential factors. Studies have shown that green spaces mediate older adults' mental health through relative deprivation. With the increase of green space, older adults' sense of relative deprivation was weakened, which indirectly improved their mental health status. Relative deprivation means that individuals or groups realize that they are in a disadvantageous position through comparison and think that they deserve better treatment, which leads to anger, resentment, anxiety and other negative emotions (85), and has adverse impact on mental health (86). For older adults, relative deprivation is associated with depression and low cognitive function (87). The existing evidence has shown that relative deprivation affects older adults more than middle-aged people (24, 57). So far, in the research on green space and mental health, academic circles have paid more attention to the differences caused by absolute deprivation, but less research on the role played by relative deprivation. Therefore, we innovatively consider the mediating role of relative deprivation in studying the relationship between green space and mental health in older adults. According to social comparison theory, individuals interacting with others with similar social status can help alleviate mental stress and cognitive inconsistency (88, 89). As an important public resource, urban green space is affected by the degree of absolute deprivation, and individuals with lower absolute deprivation can get more green space resources (47–50). As a result, individuals with different socio-economic status have differences in acquiring green space resources. The improvement of green space coverage can help improve the accessibility of green space and alleviate environmental inequality (90). Therefore, the increase in urban green space may help to narrow the difference in green space resources accessed between relatively low-status older adults and relatively high-status older adults. The contrast between the green resources subjectively available to older adults and those available to the surrounding groups may be even less obvious. Thus, it is helpful to reduce the comparison of older adults in terms of access to green space resources, relieve the pressure on older adults and their dissatisfaction with social inequity, and promote improvement in the mental health level of older adults. Earlier empirical studies have indicated that in the social environment of smaller comparison, individuals with lower status may face less obvious contrast, bear less psychological pressure, and have better mental health status (91). At the same time, in the research on the relationship between green space and wellbeing, it is also shown that relative deprivation partially mediates the relationship between green space and wellbeing (92). Wellbeing is closely related to mental health. This study confirms these early findings to some extent.

Secondly, this study also found that physical activity played an important role in the relationship between green spaces and mental health among older adults. This is consistent with the conclusions of most current studies (43, 93). Urban green spaces are the primary sites for physical exercise and activity. They provide open, healthy, and active outdoor spaces for adults, which helps enhance their physical activity abilities (94). Physical activity is important for older adults to remain healthy. Studies have shown that regular physical exercise in green spaces can greatly reduce health risks associated with cardiovascular diseases, respiratory diseases, hypertension, paralysis, diabetes, and other chronic diseases (38). Simultaneously, higher levels of physical activity are significantly associated with lower rates of mental illness, and appropriate physical activity can help reduce the risk of depression and anxiety in older adults (95), improve memory function (96), and alleviate cognitive impairment (97). Therefore, improving urban green space coverage can help improve older adults' physical activity and reduce the risk of chronic diseases and mental illness, thus improving older adults' mental health. Further, this study hypothesized that urban green spaces enhance the social trust of older adults and are thus associated with their mental health; however, the results showed that there was no significant mediating effect between social trust and green spaces and older adults' mental health. The current research mainly discusses the relationship between trust and green spaces based on trust-related indicators. For example, interpersonal trust in social capital plays a mediating role in alleviating loneliness in older adults through green space (98). But the role of social trust indicators in the relationship between green space and mental health has been less explored. More from the social cohesion, social capital, and other aspects to explore the relationship. The benefits of green spaces for older adults should be further explored (8, 14, 98, 99).

Considering the interwoven relationship between potential intermediaries, this study suggests that urban green spaces indirectly benefit older adults' mental health through relative deprivation (physical activity) and relative deprivation (with social trust). Both pathways were consistent with the expected results. The improvement of green spaces can help reduce the negative impact of relative deprivation on older adults and encourage them to carry out physical activities, thus improving mental health. There was a positive correlation between relative deprivation and physical activities. On the one hand, deprived people are less likely to be physically active than privileged people (100). On the other hand, relative deprivation disrupts the ability of older adults to age successfully and reduces healthy behaviors in older adults, including physical activity (16). The psychological pathway that intensifies social comparison is associated with individual physical activity (58). As for green space—relative deprivation—social trust—mental health pathway, relative deprivation is associated with relevant indicators of social trust. First, lower subjective social status predicts higher perceptions of relative deprivation, which in turn predicts higher interpersonal distrust (101). Individuals with lower subjective status tend to believe that they have less access to resources, and the perception of limited resources can be a threat to interpersonal communication and contribute to mistrust (102). Second, social trust is an important factor affecting emotional health; social trust can help reduce the risk of depression among older adults (103). Higher levels of social trust contribute to higher life goals and self-realization in older adults. Additionally, a high level of mental health among older adults depends on a high level of social trust and support (99). Therefore, a possible explanation is that green spaces can improve older adults' sense of trust in society by reducing the negative impact of relative deprivation on interpersonal relationships, thus affecting their mental health.

This study has some limitations. First, the data selected for this study were cross-sectional. In the statistical collection of cross-sectional data, there may be missing variables or unobservable differences between individuals. Second, owing to data limitations, this study did not consider attributes such as accessibility, quality, type, and subjective perception of green spaces. Although the coverage rate of green space is also an important indicator of urban green space, the lack of other green space attributes affects the final results of the study to some extent. Simultaneously, limited by the original data of the study sample, only a single indicator was selected to measure relative deprivation. Although relative deprivation can be explained to a certain extent, the ideal state of multidimensional measurement has not been reached, which may affect the accuracy of the results. More complex tests will be required in future studies and analyses.

## 6 Conclusion

This study employed SEM model, using statistics from the nationally representative 2018 CLDS database, to examine the relationship between urban green space rates and older adults' mental health in Chinese urban settings, with a particular focus on the multiple mediating roles of relative deprivation, physical activity, and social trust. The SEM results indicated that urban green spaces were positively associated with older adults' mental health. This suggests that having more greenery in built-up urban areas can improve older adults' mental health. The results of the mediation analysis showed that green spaces can enhance the sense of relative deprivation and physical activity, which, in turn, affects older adults' mental health. There was no evidence that social trust directly mediates the relationship between green spaces and mental health. Further, relative deprivation can mediate the effect of green spaces on mental health by promoting physical activity, social trust, and ultimately, mental health among older adults.

The results of this study are of great significance for promoting the construction of healthy living cities and ensuring healthy aging in China. First, green space can improve the mental health of older adults. The government should implement scientific urban environmental intervention measures to enhance the construction of green space in the urban. Secondly, from the perspective of the role of relative deprivation in the connection between green space and the mental health of older adults, it is very important to promote the environmental equity of urban green space. In the long-term development of the urban, the use of urban planning means to build more fair, safe and comfortable urban public green space and narrow the difference of green space resources obtained by older adults with different social and economic status plays an important role in improving the mental health level of older adults. But in the short term, it is not realistic to increase the green space in the urban. Therefore, other ways are recommended to make the health benefits of green space better reach older adults. Emphasis should be placed on improving the quality of existing green Spaces, especially those in poorer urban environments, taking into account the actual needs of older adults to use green Spaces, such as building sound barrier-free facilities and eliminating unnecessary height differences. At the same time, the government or all sectors of society can make use of urban green Spaces to carry out social activities for older adults, encourage older adults to engage in physical exercise and social interaction in green Spaces, and increase the opportunities for older adults to contact the natural environment in their daily lives. For example, gardening activities for older adults are carried out in urban public green Spaces to provide an interesting and enjoyable gardening experience for older adults. To increase older adults' contact with natural landscapes, plants and sounds, in order to reduce the generation of negative emotions and improve their mental health (104). In addition, according to our research results, physical activities in green areas are conducive to the maintenance of good mental health of older adults, and activities such as aerobics, dancing and walking can be organized in green spaces for older adults. Moreover, social interaction in green Spaces contributes to positive psychological changes. Carrying out social activities for older adults in green space, such as literary performances and chess games, is conducive to increasing the social interaction opportunities of older adults, promoting the improvement of older adults' sense of social trust, and reducing the negative mental health status of older adults. Finally, the good quality of urban green space and the development of older adults' activities cannot be separated from a sound green space operation, maintenance and management system. Effective management of urban green space plays an important role in promoting the long-term benefit of green space to the mental health of older adults.

## Data Availability

The original contributions presented in the study are included in the article/supplementary material, further inquiries can be directed to the corresponding author.
